# The hippocampus extrapolates beyond the view in scenes: An fMRI study of boundary extension

**DOI:** 10.1016/j.cortex.2012.11.010

**Published:** 2013-09

**Authors:** Martin J. Chadwick, Sinéad L. Mullally, Eleanor A. Maguire

**Affiliations:** Wellcome Trust Centre for Neuroimaging, Institute of Neurology, University College London, London, UK

**Keywords:** Hippocampus, Scenes, Boundary extension, fMRI, Parahippocampal cortex

## Abstract

Boundary extension (BE) is a pervasive phenomenon whereby people remember seeing more of a scene than was present in the physical input, because they extrapolate beyond the borders of the original stimulus. This automatic embedding of a scene into a wider context supports our experience of a continuous and coherent world, and is therefore highly adaptive. BE, whilst occurring rapidly, is nevertheless thought to comprise two stages. The first involves the active extrapolation of the scene beyond its physical boundaries, and is constructive in nature. The second phase occurs at retrieval, where the initial extrapolation beyond the original scene borders is revealed by a subsequent memory error. The brain regions associated with the initial, and crucial, extrapolation of a scene beyond the view have never been investigated. Here, using functional MRI (fMRI) and a classic BE paradigm, we found that this extrapolation of scenes occurred rapidly around the time a scene was first viewed, and was associated with engagement of the hippocampus (HC) and parahippocampal cortex (PHC). Using connectivity analyses we determined that the HC in particular seemed to drive the BE effect, exerting top–down influence on PHC and indeed as far back down the processing stream as early visual cortex (VC). These cortical regions subsequently displayed activity profiles that tracked the trial-by-trial subjective perception of the scenes, rather than physical reality, thereby reflecting the behavioural expression of the BE error. Together our results show that the HC is involved in the active extrapolation of scenes beyond their physical borders. This information is then automatically and rapidly channelled through the scene processing hierarchy as far back as early VC. This suggests that the anticipation and construction of scenes is a pervasive and important aspect of our online perception, with the HC playing a central role.

## 1 Introduction

In the natural world, what we see is always embedded within a wider context. As such, we never perceive what is in front of our eyes in complete isolation, but instead an object is perceived as part of a visual scene, and each scene as one of an infinite set of related scenes that somehow form a continuous sense of space and place. A central tenet of perception is that visual input is necessarily limited and ambiguous. The brain overcomes this by making predictions about the likely content of the external world, extrapolating beyond the information that is directly available through the senses (Gregory, 1968, 1980; Friston, 2010). This is exemplified by a phenomenon known as ‘boundary extension’ (BE), whereby people reliably remember seeing more of a scene than was present in the physical input, because they extrapolate beyond the borders of the original stimulus (Intraub and Richardson, 1989).

**Fig. 1:**
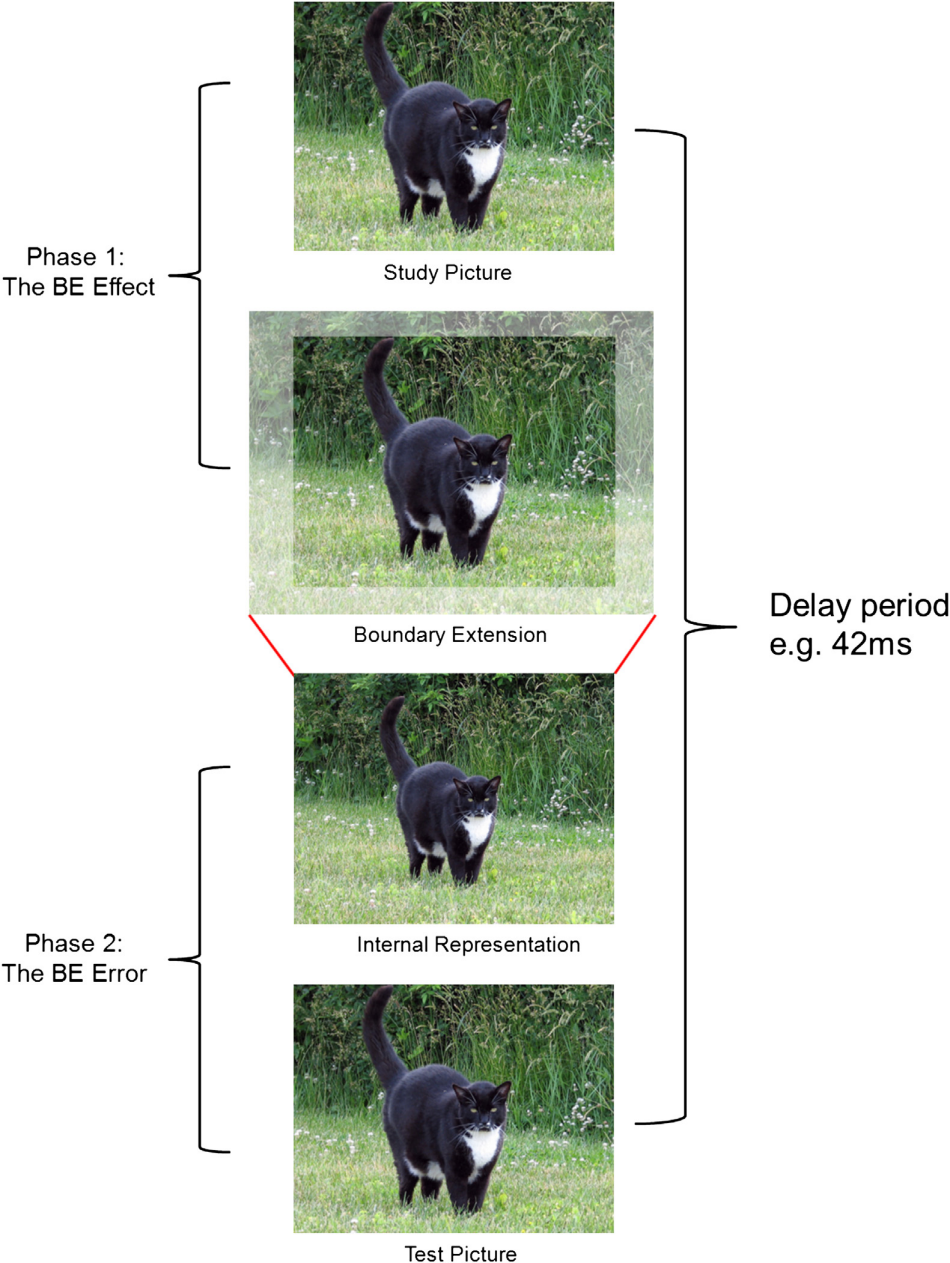
The phenomenon of BE. When we see a picture of a scene (top panel), we automatically extrapolate beyond the physical edges of that scene (second panel). This active extension of the scene is the ‘BE effect’. When the scene is no longer present, the extended content and context beyond the boundaries become incorporated into our internal representation of the scene (third panel). Thus, in Phase 2, when exactly the same picture is presented at test (fourth panel), we compare the now extended internal representation to the test picture, leading to a perception that the test picture is ‘closer’ than the original study picture, even though they are identical. This memory error is the ‘BE error’.

BE occurs across a variety of testing conditions including recognition, free recall, both visually and haptically (Intraub, 2004, 2012). It is apparent in all populations sampled including adults (Intraub and Richardson, 1989; Seamon et al., 2002), children (Seamon et al., 2002; Candel et al., 2004), and even babies (Quinn and Intraub, 2007). Importantly, BE only occurs in response to scenes, and not isolated objects (Intraub et al., 1998; Gottesman and Intraub, 2002). It is thought to comprise a two-stage process (Fig. 1); the first stage involves the active extrapolation of the scene beyond its physical boundaries, and is constructive in nature. This occurs because when we initially encounter a scene, we are not limited to the direct sensory input entering the retina, but also have access to an automatically constructed and implicitly maintained representation of the scene. This constructed representation extends beyond the borders of the physical scene, and provides a global framework into which it can be rapidly embedded (Intraub, 2012). This process supports our experience of a continuous and coherent world, despite it being amassed from discontinuous sensory input, and is therefore highly adaptive.

The extended scene becomes incorporated into our internal representation of that scene, and this persists when the scene is no longer present. The second phase of BE occurs at retrieval, where the extrapolation beyond the original scene borders that occurred in the first phase is revealed by a subsequent memory error. Specifically, if presented with exactly the same scene a second time, people frequently judge the scene on this occasion to have less background, making it appear to be closer-up than the first scene. The fact that the studied view need only be absent for as little as 42 msec for BE to be apparent (Intraub and Dickinson, 2008) underscores the online and spontaneous nature of this effect. The first stage of BE, involving the active extrapolation of the scene beyond the boundaries, we hereafter refer to as the BE effect to differentiate it from the subsequent memory error, which we call the BE error.

The BE effect captures something automatic and fundamental about our interaction with the world yet its neural substrates have not been well-characterised. The only neuropsychological study of BE was conducted recently by Mullally et al. (2012), who examined BE in patients with selective bilateral hippocampal damage and concomitant amnesia. Notably, these patients were also impaired at constructing fictitious and future scenes and events in the imagination (see also Hassabis et al., 2007; Rosenbaum et al., 2009; Andelman et al., 2010; Race et al., 2011). The extrapolation of scenes beyond the view depends on intact scene construction ability (Hassabis and Maguire, 2007, 2009), suggesting that BE should be reduced in such patients. This is indeed what Mullally et al. (2012) found, with BE significantly attenuated compared to matched control participants across a variety of BE paradigms leading to the conclusion that the hippocampus (HC) supports the internal construction of scenes and also extended scenes when they are not physically in view.

Only one functional magnetic resonance imaging (fMRI) study has examined the neural correlates of BE, using a region-of-interest (ROI) approach focused on two scene-relevant brain areas, the posterior parahippocampal cortex (PHC) and retrosplenial cortex (RSC) (Park et al., 2007). The aim of their study was not to investigate activity relating to the initial extension of a scene during the first presentation (the BE effect), but instead was to examine neural adaptation (i.e., attenuation in the neural response with repeated presentation of a stimulus – see Grill-Spector et al., 2006) on presentation of the second scene. They found that both PHC and RSC demonstrated adaptation effects consistent with the subjective perception of scenes rather than the physical reality. The results of this study suggest that these scene-relevant regions are sensitive to the output of BE at the BE error stage. The findings from Park et al. (2007), however, do not allow any conclusions to be drawn about the brain areas involved in the initial stage of automatic extrapolation beyond the view of scenes, the BE effect itself.

The current study therefore had three aims. First, using fMRI in healthy participants we focussed specifically on the BE effect, the initial stage of scene extrapolation, in order to ascertain how this is instantiated in the brain, and in so doing to throw further light on this highly adaptive process. Second, we sought to establish if the HC was engaged during BE, in line with the findings of Mullally et al. (2012). Specifically, we wondered if the HC would be involved in the initial stage of scene extrapolation. If so, this automatic and implicit role in constructing and representing unseen aspects of scenes would provide further insights into the nature of hippocampal processing. Third, as well as the HC, and given the findings of Park et al. (2007), we were also interested to know if areas such as PHC would be engaged. In particular we wanted to gain new insights into the flow of scene-related information by assessing the effective connectivity between implicated brain regions during the initial scene extrapolation stage of BE.

**Fig. 2:**
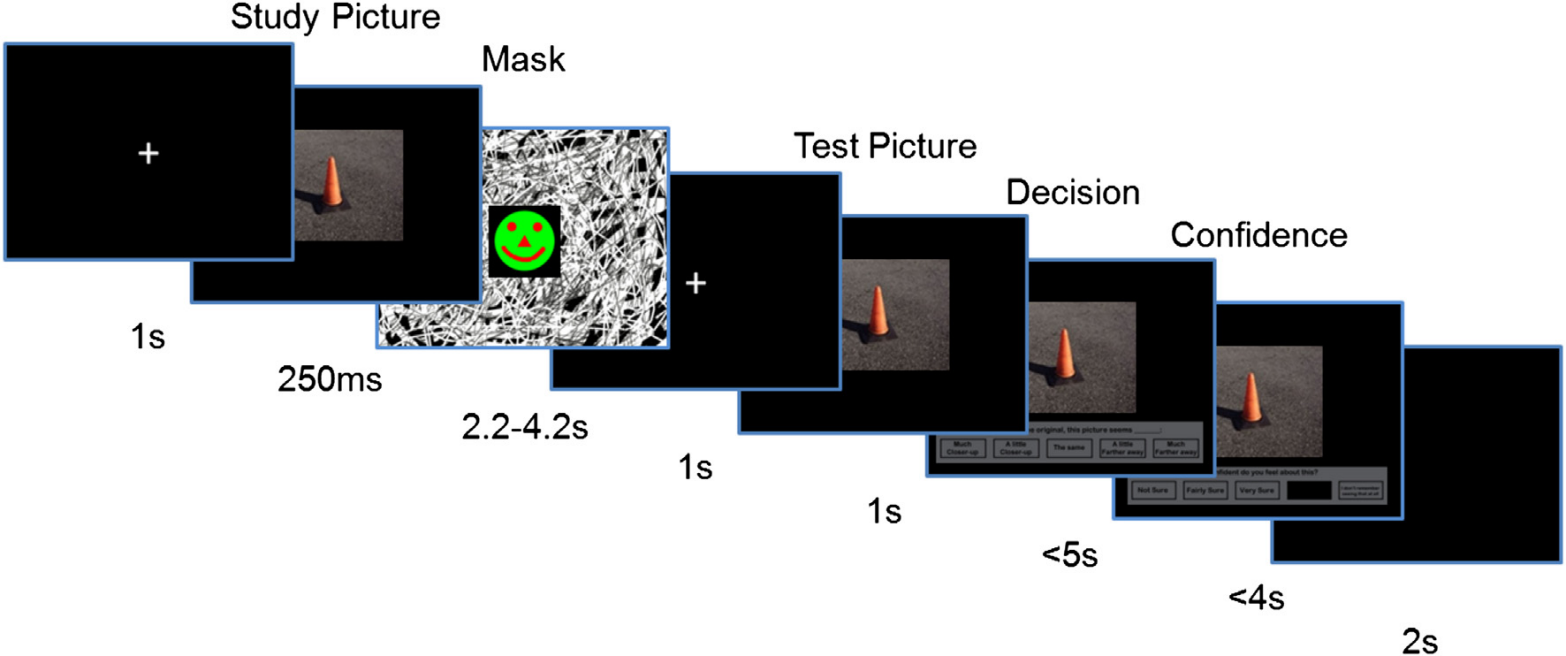
An example of a trial during the fMRI experiment.

In order to do this, we used a modified version of a classic BE paradigm, known as the rapid serial visual presentation (RSVP) task (Fig. 2), where on each trial a picture of a scene was presented briefly, followed by a visual mask (Intraub et al., 1996; Intraub and Dickinson, 2008; Mullally et al., 2012). After a short interval (and unbeknownst to the participants) exactly the same scene was presented for a second time, and the participant was required to decide whether the second scene appeared to be exactly the same as the first (the correct answer), closer or further away. On a high proportion of trials in this task (e.g., ∼60% in Mullally et al., 2012), healthy participants rate the second picture as closer-up than the first picture, thus exhibiting BE (Intraub et al., 1996).

To investigate neural activity related specifically to the BE effect, we capitalised on the fact that in the RSVP task BE does not happen on every trial. This allowed us to compare trials where BE occurred to those where it did not. By focussing exclusively on the first occasion that each scene was viewed, we could compare the activity elicited during the first scene presentation in trials which subsequently led to a BE error and those first presentations of scenes which did not lead to a BE error. Regions involved in the automatic construction of extended scenes should show increased activity on trials where the BE effect occurred compared to those where it did not.

## 2 Methods

### 2.1 Participants

Thirty healthy right-handed adults [15 females; mean age 22.0 years; standard deviation (SD) 2.88; range 19–28 years] participated in the experiment. All had normal or corrected-to-normal vision and gave informed written consent to participation in accordance with the local research ethics committee. Participants were naïve to the concept of BE, and it was not mentioned at any time during the experiment.

### 2.2 Task and procedure

During a pre-scan training period participants were instructed in the task requirements and performed practice trials using material that was not included in the main scanning experiment. They were informed that they would be viewing a scene that would be presented twice, and that when the scene was presented the second time it might appear to be exactly the same, closer-up or further away than when first viewed. The aim of the task was simply to decide on each trial whether the second scene appeared to be closer, further away, or the same. During subsequent fMRI scanning participants completed 60 trials of the task, presented in a randomised order, with a different scene used on each trial. In a post-scan debriefing session, each participant confirmed they had complied with the task instructions and had made the intended responses.

At the start of each trial a central fixation cross appeared, indicating that the trial was starting (Fig. 2). After 1 sec a scene was briefly presented in the centre of the screen for 250 msec. This was then concealed with a dynamically changing visual noise mask which lasted for 200 msec (Intraub and Dickinson, 2008). This was followed by a static visual noise mask presented for a variable period of 2, 3 or 4 sec. The length of this “jitter” was pseudo-randomised across trials. The purpose of this jittered period was to create separable neural signals for both the first and second scene presentations (Dale, 1999), although the key comparison of interest here was in fact between different types of first scene presentations. Jittering is a common approach in event-related fMRI studies, used to de-correlate the blood oxygenation level-dependent (BOLD) signal associated with two events that are presented close to one another in time, such as the two scenes presented in this study. At the end of the jitter period a central fixation cross appeared for 1 sec, followed by the scene presented once again and in the same location. After 1 sec the scene was joined by a set of options which appeared underneath the picture.

Participants were first provided with a set of five possible responses indicating that the second picture appeared to be “much closer-up” than the first picture, that it was “a little closer-up”, that it was “the same” (the correct answer), that it was “a little further away”, or that it was “much further away”. They were allowed up to 5 sec to select one option using a five-button scanner-compatible button-box using their right hand. Once they had made their response (or if they had failed to respond within 5 sec), a second set of options appeared, requiring the participant to make a confidence judgement regarding their decision. The choices indicated that the participant was “not sure” of their response, that they were “fairly sure”, or that they were “very sure”; participants were allowed up to 4 sec to select one option. They were also given the option to press a button to indicate that they did not remember seeing the first picture at all. This was included given the rapid presentation of the first scene and to allow for the fact that a participant may occasionally miss a scene due to momentary inattention or protracted blinking. Any trials on which a participant provided this response were discarded from the subsequent analysis, as were trials on which participant failed to provide a response to either of the ratings [mean number of excluded trials 1.53 (SD 2.5)]. Participants then had 2 sec to rest before the start of the next trial.

### 2.3 Behavioural analysis

Following the scoring procedure of Intraub and Richardson (1989), each response was scored from −2 to 2 where −2 meant “much closer-up”, −1 meant “a little closer-up”, 0 meant “the same”, 1 meant “a little further away”, and 2 meant “much further away”. The mean score across all trials was calculated for each participant, providing an overall BE score. This score indicates the degree of bias towards one response over another. If participants show no bias in response, the score will be 0. However, if they display a BE effect, the score will be negative, due to the greater number of closer responses. In order to determine whether the group of participants as a whole displayed a significant BE effect, we compared the BE scores to 0 using a *t*-test. We also performed a second analysis where we investigated the proportion of each response type (Closer, Same, Further), ignoring the degree of subjective distance (i.e., whether it was “much” or “a little” further/closer). For this analysis we calculated the percentage of response trials falling into each of the three categories for each participant, and compared them using a one-way analysis of variance (ANOVA).

### 2.4 MRI scanning

MRI data were collected using a 3 T Magnetom Allegra head-only MRI scanner (Siemens Healthcare, Erlangen, Germany) operated with the standard transmit-receive head coil. Functional MRI data were acquired in one session with a BOLD-sensitive T2*-weighted single-shot echo-planar imaging sequence which was optimised to minimise signal dropout in the medial temporal lobe (MTL) (Weiskopf et al., 2006). The sequence used a descending slice acquisition order with a slice thickness of 2 mm, an interslice gap of 1 mm, and an in-plane resolution of 3 × 3 mm. Forty eight slices were collected covering the entire brain, resulting in a repetition time of 2.88 sec. The echo time was 30 msec and the flip angle 90°. All data were acquired at a −45° angle to the anterior–posterior axis. In addition, field maps were collected for subsequent distortion correction (Weiskopf et al., 2006). These were acquired with a double-echo gradient echo field map sequence (TE = 10 and 12.46 msec, TR = 1020 msec, matrix size 64 × 64, with 64 slices, voxel size = 3 mm^3^) covering the whole head. After these functional scans, a 3D MDEFT T1-weighted structural scan was acquired for each participant with 1 mm isotropic resolution (Deichmann et al., 2004).

### 2.5 Image pre-processing

Neuroimaging data were analysed using SPM8. The first six functional volumes were discarded to allow for T1 equilibration (Frackowiak et al., 2004). The remaining functional volumes were spatially realigned to the first image of the series, and distortion corrections were applied based on the field maps using the Unwarp routines in SPM (Andersson et al., 2001; Hutton et al., 2002). Each participant's structural scan was then co-registered to a mean image of their realigned, distortion-corrected functional scans. The structural images were segmented into grey matter (GM), white matter (WM), and cerebral spinal fluid using the New Segment tool within SPM8. The DARTEL normalization process was then applied to the GM and WM segmented images, which iteratively warped the images into a common space using nonlinear registration (Ashburner, 2007). Using the output of this nonlinear warping process, all functional and structural images were normalised to MNI space using DARTEL's ‘Normalise to MNI’ tool. The functional images were smoothed using a Gaussian kernel with full-width at half maximum of 8 mm.

Structural MRI scans were analysed using voxel-based morphometry (VBM; Ashburner and Friston, 2000, 2005) implemented in SPM8, employing a smoothing kernel of 8 mm full-width at half maximum. For *a priori* ROIs (HC, PHC and RSC – see Section 2.7), we applied a statistical threshold of *p* < .001 uncorrected for multiple comparisons. For the rest of the brain, we employed a family-wise error (FWE)-corrected threshold of *p* < .05. We searched for structural correlates of individual differences in BE, and found no significant effects in the MTLs, or elsewhere in the brain.

### 2.6 Neuroimaging analyses

Statistical analysis of the fMRI data was applied to the pre-processed data using a general linear model. The primary analysis involved a comparison of activity elicited by the first scene presentation on trials where BE occurred and those first presentation trials where it did not. To do this, we used each participant's behavioural data in order to divide the trials into those where BE occurred (all trials where the second scene was judged to be closer than the first – the BE condition), and those where it did not occur (the Null condition). The Null condition consisted of trials where the second scene was judged to be the same or further away than the first, as in both cases BE did not occur. By pooling across both types of Null trial in this way, we increased the power of the analysis. We used a stick function to model the onset of each first scene presentation, dividing the trials into two conditions based on the subsequent behavioural choice data, thus creating a BE regressor and a Null regressor. These stick functions were convolved with the canonical haemodynamic response function and its temporal derivative to create the two regressors of interest. We also used a stick function to model the second scene presentations, dividing them into BE and Null conditions, which were included as regressors of no interest. The decision and confidence rating periods were modelled as boxcar functions with variable length, depending on the participant-specific response times, and were included as regressors of no interest. Subject-specific movement parameters were also included as regressors of no interest. Participant-specific parameter estimates (*β* values) were calculated at each voxel across the brain. The parameter estimates were then entered into a second level random effects analysis, where one-sample *t*-tests were applied to every voxel. Initial statistical thresholding was applied using a threshold of *p* < .001, uncorrected for multiple comparisons. Activations were considered to be statistically significant only if they survived FWE correction at either the peak or cluster level. For *a priori* anatomical ROIs, FWE correction was applied using small volume correction (Frackowiak et al., 2004) within pre-defined anatomical masks (see Section 2.7).

Although not our primary interest, given the results of Park et al. (2007), we also looked for adaptation effects. Here we contrasted trials where the two scenes were perceived to be the same with those that were perceived to be different (including both closer and further away), despite the stimuli being physically identical during any one trial. The trials were divided into these two conditions for modelling both the first and second scene presentations. In all other respects, the experimental design was identical to that described above. We first used a whole-brain analysis to localise regions which displayed an overall adaptation effect between the first and second scene presentations, regardless of condition. We then conducted more in-depth adaptation analyses using ROIs, as described below. The statistical thresholds were identical to those described above.

### 2.7 ROIs

Given the (limited) previous literature on the functional neuroanatomy of BE, our *a priori* hypothesis was that the HC would be involved in the BE effect, and that the PHC and RSC might also be implicated. Each of these ROIs was manually defined on the normalised group average T1-weighted structural MR image using the Duvernoy anatomical atlases for guidance (Duvernoy, 1999, 2005). These anatomical ROIs were used in MarsBar (http://marsbar.sourceforge.net) analyses, where a finite impulse response (FIR) model (Dale, 1999; Ollinger et al., 2001) was fitted to the data in order to probe the time-course of responses in ROIs. Four time-windows of 2 sec each were modelled, time-locked to the onset of the first scene presentation. These ROIs were also used for small volume correction within SPM. Based on the whole-brain adaptation analysis described above, we determined that early visual cortex (VC) was a target for further ROI-based analyses. We therefore established a VC ROI using a contrast that was orthogonal to the adaptation analysis (i.e., all scenes presented on the first trial only compared to the implicit baseline). This ROI was used for further adaptation analyses, and for the HC–VC dynamic causal modelling (DCM) analysis described below.

### 2.8 DCM

The anatomical ROIs were also used for DCM, a Bayesian model comparison method which involves creating various plausible models of the task-dependent effective connectivity between pre-specified brain regions (Friston et al., 2003; Stephan et al., 2010). Once fitted, the evidence associated with each model can be compared in order to determine which is the most likely (or ‘winning’) model. We were interested in investigating the modulation of effective connectivity elicited by the presentation of the first scene on trials where BE occurred, and in order to do this we created a simplified design matrix for the DCM analysis, consisting of two regressors. The first modelled the onset of all first scene presentations, and the second modelled the first scene presentations on trials where BE occurred. Two separate DCM analyses were conducted, in each case investigating the connectivity between two ROIs (HC and PHC in one set of models, HC and VC in the second). DCM10 was used for these analyses, and in both cases the two ROIs were considered to have reciprocal average connections (the A matrix), with the visual input (the C matrix) stimulating the PHC in the first analysis and VC in the second. For both analyses there were three different models based on altering the modulatory connections (the B matrix), allowing the modulation to affect the “backward” connection (from HC back to either PHC or VC), the “forward” connection, or both directions (“bidirectional”). Separate analyses were conducted in both hemispheres, and used a random effects Bayesian model comparison method to determine which was the winning model (Stephan et al., 2009, 2010). This results in an exceedance probability estimate for each model, which describes how likely that model is compared with any other model. The model with the highest exceedance probability is considered to be the winning model.

## 3 Results

### 3.1 Behavioural evidence for BE

**Fig. 3:**
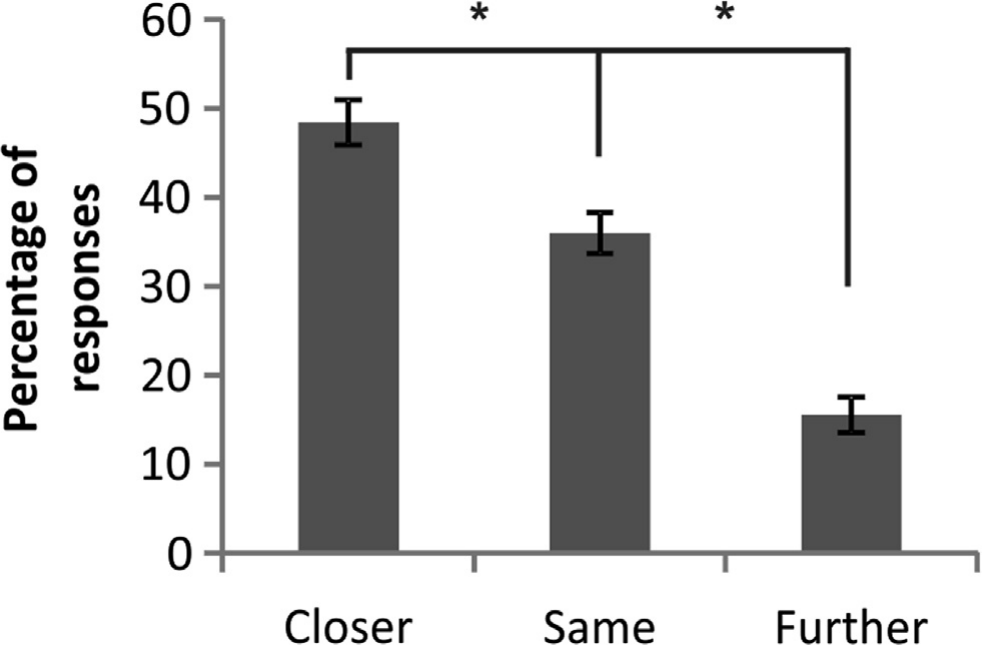
Behavioural responses. The percentage of trials perceived as “Closer”, “the Same”, and “Further Away” was calculated for each individual. The group mean percentage for each response type is displayed here. The proportion of “Closer” responses was significantly greater than each of the other responses, demonstrating a BE effect.

The RSVP task resulted in BE with a mean average BE score of −.40 (SD .26). A negative score indicates a bias towards responding “Closer”, consistent with a BE effect. A *t*-test comparing scores against 0 demonstrated that this behavioural effect was highly significant (*t* = −8.58, *p* < 10^−9^). In a second analysis, we calculated the percentage of each categorical response type (Closer, Same, Further) for each participant (displayed in Fig. 3). A one-way repeated-measures ANOVA demonstrated that there was significant variation in response across these three conditions (*F* = 34.65, *p* < 10^−32^). Post-hoc *t*-tests revealed that the percentage of Closer responses was significantly greater than both the Further (*t* = 10.17, *p* < 10^−14^) and Same responses (*t* = 3.61, *p* = .0006), consistent with BE. Together, both analysis methods reveal a robust behavioural BE effect.

Importantly, despite the strong overall BE effect and as is usual in this task, BE was not apparent on all trials for any of the participants; the mean proportion of trials on which a participant produced a BE error was 48% (SD 14%). This provided an even division of the data for the main neuroimaging contrast between first presentations of scenes where BE occurred and those where it did not.

Because a jittered inter-stimulus interval was used in this study, we tested whether this variation in time affected the behavioural responses. We calculated a BE score separately for each inter-stimulus interval (ISI) (2, 3 and 4 sec) for each participant. We then tested for any differences between these levels of jitter using a one-way repeated-measures ANOVA. No significant effect was found, indicating that the different levels of jitter did not impact significantly on the BE effect (*F* = .60, *p* = .55).

We also investigated whether there were systematic differences in BE across the scene stimuli. We calculated the cross-participant SD for each scene (mean SD = .91, SD of the SD = .10, range of the SD = .67–1.11) and found substantial variation across participants for each item, suggesting there was no consistent item-level effects on BE. To determine whether there were any specific scenes which had a particularly strong (or weak) BE effect compared to the others, in a second analysis we looked at the set of mean BE scores for each of the 60 scene stimuli. If any individual scenes were exerting a consistently strong or weak BE effect, then the mean BE scores should be particularly high (or low) compared to the whole distribution. In other words, they should show up as an outlier (three SDs or more from the mean). This was not the case for any of the scenes, and the maximum SD was only 2.19 from the mean. This suggests that no individual scenes exerted a systematically strong or weak BE effect.

### 3.2 Brain areas involved in the initial extrapolation of scenes

**Fig. 4:**
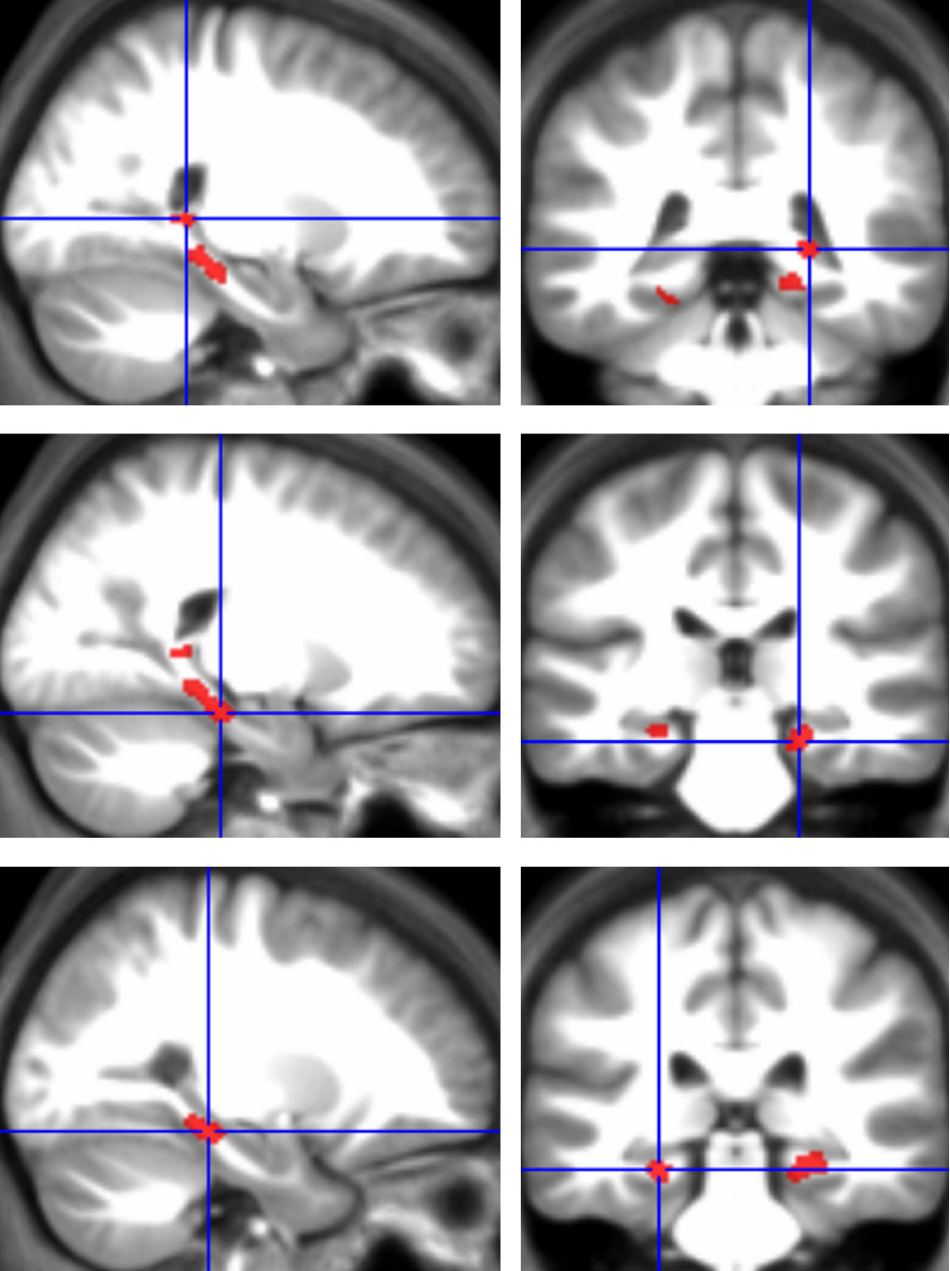
Neural correlates of the BE effect. Trials on which BE occurred were compared to those where it did not, focussing specifically on activity evoked by the first scene presentation. Several areas within the MTL showed increased engagement during the extrapolation of scenes beyond the view. Results are displayed on the group average structural MRI scan in the sagittal plane on the left, and the coronal plane on the right, with the crosshairs centred on the peak of the activation in each case. The top panel displays the activation in right posterior HC, the middle panel the right PHC activation, and the bottom panel activation in the left MTL spanning both HC and PHC. For display clarity, activity is thresholded at *p* = .005 uncorrected, and with a MTL mask.

We conducted a whole-brain fMRI analysis contrasting activity on first presentation trials where BE subsequently occurred to those first presentation trials where it did not (scenes judged to be the same or further away). We focussed on activity evoked by the first scene presentation because this is the point at which the BE effect is proposed to take place. This analysis (Fig. 4) revealed significant activation in the right posterior HC (peak coordinate 24, −39, 3; *Z* = 3.42; cluster size 20), right PHC (21, −27, −18; *Z* = 3.71; cluster size 46), and a significant activation extending across both left posterior HC and left PHC (−26, −31, −14; *Z* = 3.45; cluster size 35). No other significant activations were apparent elsewhere in the brain, including the RSC (a region previously implicated in BE – Park et al., 2007), indicating that this effect was localised to the MTLs.

### 3.3 Time-course of responses

**Fig. 5:**
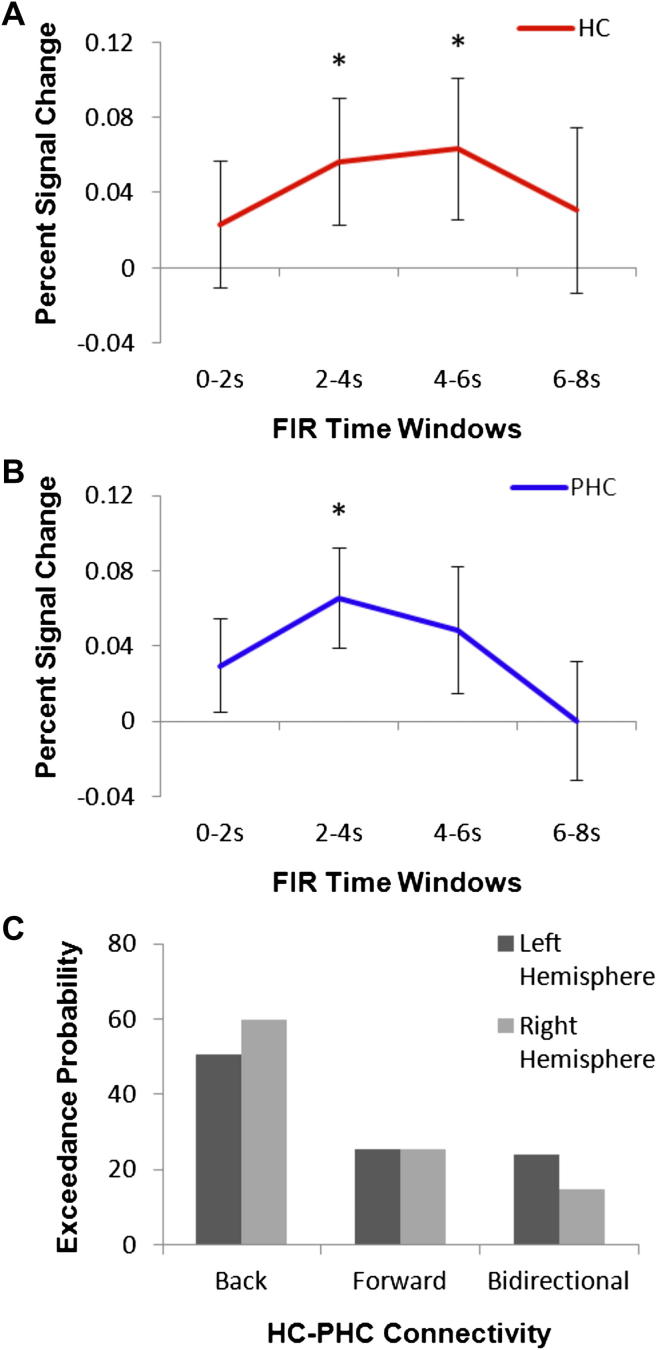
FIR analysis was used to investigate the time-course of responses in the HC (A) and PHC. (B). In each case the increase in activity for trials where BE occurred compared to those where it did not are plotted, with standard error bars. The different FIR time-windows are displayed on the *x* axis, and percent change in fMRI BOLD response on the *y* axis. For both regions a significant increase in activation as early as 2–4 sec following the presentation of the first scene was apparent (which was before the presentation of the second scene). Furthermore, given that the BOLD response lags behind stimulus presentation with a peak of 6 sec, this reflected a rapid response to the first scene. (C) The results of the DCM model comparison analysis. This plot displays the exceedance probability on the *y* axis, which describes how likely each model is compared to any other model. This is shown for the three possible models. The ‘back’ model was the winner in both hemispheres, suggesting that the HC is the driving force behind the BE effect, and influences PHC.

In order to assert that the MTL activity observed here reflected the active extrapolation of scenes, it was important to establish that the responses were indeed evoked by the first scene presentation. We therefore examined the time-course of activity within each of the activated regions (ROIs were anatomically defined – see Section 2.7) using a FIR analysis in MarsBar. This allowed us to examine the fMRI signal within specific time-windows of 2 sec each that were time-locked to the onset of the first scene presentation on each trial. This analysis revealed a significant increase in activity on trials where BE occurred as early as 2–4 sec following the first scene onset (collapsed across hemisphere: HC *t* = 2.11, *p* = .02; PHC *t* = 1.94, *p* = .03), indicating that this is an early response that likely occurred soon after stimulus onset (Fig. 5A and B). Given that the shortest delay between the onset of the first and second scene presentations was 3.45 sec (occurring on one third of the trials due to the jittered delay), we can conclude with some certainty that this effect during the 2–4 sec time-window can only be attributed to a process occurring in response to the first scene. Furthermore, given that the BOLD signal lags behind cognitive processes with a peak response at around 6 sec after stimulus presentation, this early response at 2–4 sec suggests a rapid response to the first stimulus. Due to the limited temporal resolution of fMRI, it is not possible to determine whether the signal can be attributed to a process occurring online, during perception of the scene, or shortly after the stimulus offset. Nevertheless, we can conclude that the BE-related activity occurred in response to the first scene, prior to the onset of the second scene, which was the critical question of interest here.

### 3.4 HC–PHC connectivity

These results clearly implicate both the HC and PHC in BE. Our hypothesis was that the HC plays a central role in the BE effect, because patients with damage localised to the HC show reductions in BE (Mullally et al., 2012). It was therefore important to tease apart the functional contributions of these two regions by investigating the neural dynamics occurring during the BE effect. If our hypothesis was correct, then we would expect the HC to be driving the activity of the PHC. The flow of information between these two regions was assessed using DCM (see Section 2.8), a Bayesian model comparison method in which different models of the neural dynamics are compared in order to find the most likely model of information flow in the brain (Friston et al., 2003).

For this analysis, we used a simple approach which involved investigating the connectivity between the two ROIs, the HC and PHC. We conducted this analysis separately in both hemispheres, and used a random effects Bayesian model comparison method to determine which was the winning model (Stephan et al., 2009, 2010). The winning model was the backward modulation model, in which the HC drove activity within the PHC, and this was the case for both hemispheres independently (exceedance probability for the backward model was 60% in the right, and 51% in the left hemisphere; Fig. 5C). This result suggests that the HC is the driving force behind the BE effect, which then influences activity within the PHC.

### 3.5 fMRI adaptation

**Fig. 6:**
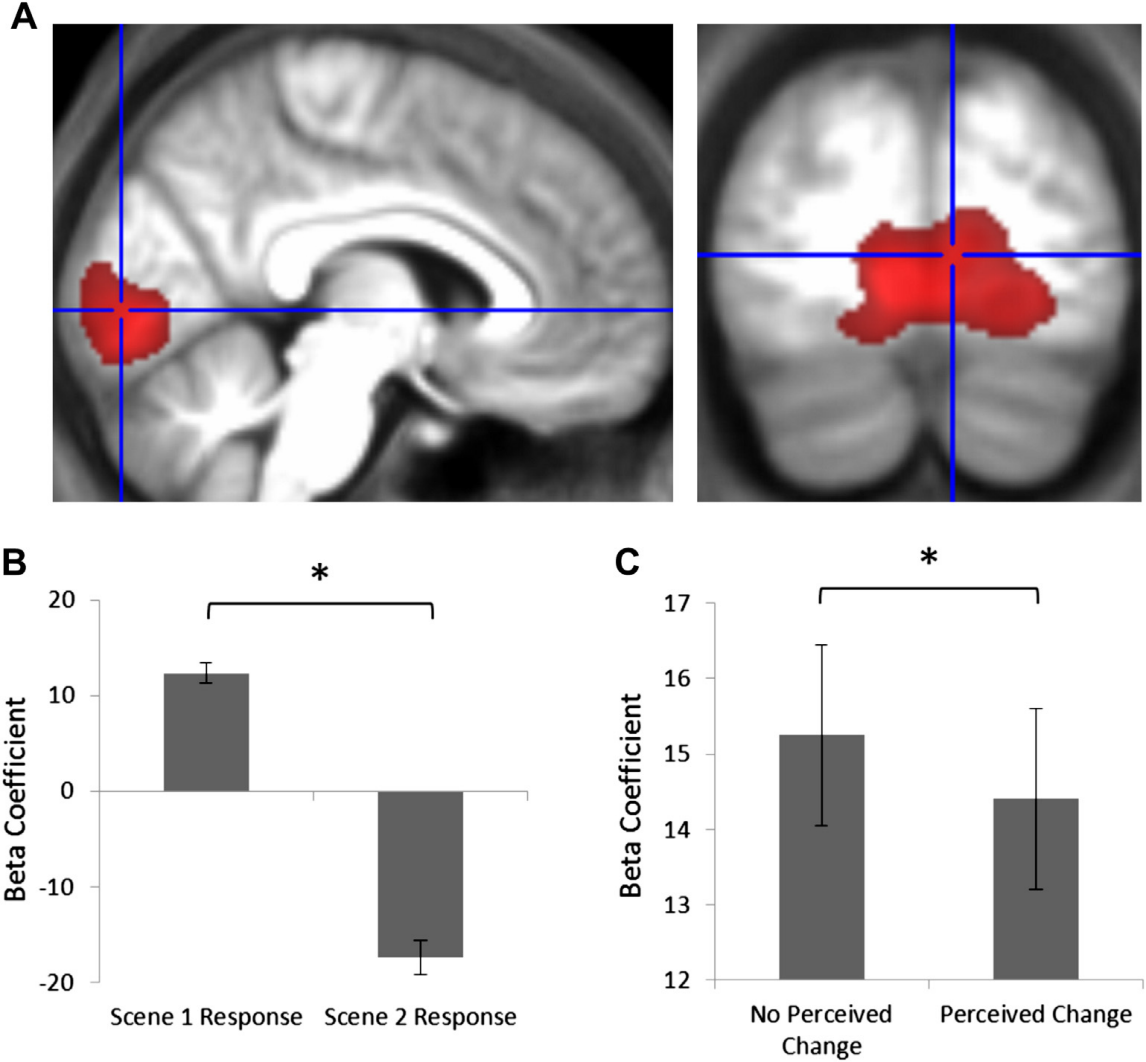
Whole-brain analysis investigating fMRI adaptation effects between the first and second presentation of scenes. The only activation was in early VC (A), displayed here at a FWE-corrected threshold of *p* < .05 on the group average structural MRI scan. The crosshair is centred on the peak of the activation. (B) The average response within this region to the first and second scene presentations, with standard error bars. This plot demonstrates that VC showed a robust adaptation effect to repeated scene presentations. The *y* axis displays the parameter estimates from the general linear model. (C) The magnitude of the adaptation effect (i.e., the amount of attenuation between first and second scene presentation) for the two conditions of interest, with standard error bars. The *y* axis displays the contrast between the parameter estimates for the first and second scenes. When participants perceived a change between the first and second scene presentation (e.g., when it appeared to be closer) there was a significant reduction in the magnitude of adaptation compared to trials where participants perceived no change between the two scenes. This was despite the fact that the two scenes in a trial were always physically identical.

Having conducted the primary analyses of interest, we were able to ask a further question with this dataset, namely, were there any changes in fMRI activity consistent with the subjective perception of scenes, similar to the approach adopted by Park et al. (2007)? fMRI adaptation is a method based on the observation that fMRI activity is attenuated with repeated presentation of a stimulus (Grill-Spector et al., 2006). To investigate this, we first searched for regions showing an overall adaptation effect in response to scenes, regardless of the behavioural response. Interestingly, the only brain region to show an overall adaptation effect was early VC (peak coordinate −6, −85, −3; *Z* = 7.62; cluster size 5128, using peak threshold of FWE *p* < .05; see Fig. 6A and B). Using MarsBar to probe the average activity in the pre-defined ROIs confirmed that none of the MTL regions displayed an overall adaptation effect in response to the scenes. In order to further investigate the adaptation effect within early VC, an ROI was established using a contrast that was orthogonal to the adaptation analysis (i.e., all scenes presented on the first trial only compared to the implicit baseline).

Having defined this ROI, we next wanted to look for evidence of *differential* adaptation effects in line with subjective perception of the scenes. MarsBar was used to extract the mean adaptation response on trials where participants perceived the second scene to be exactly the same as the first (no change in subjective perception) and those where the second scene was perceived to be different from the first (either closer or further away). If the early VC displayed responses that reflected the subjective perception of the scenes, we would expect this region to display less adaptation on trials where the scenes are perceived to be different compared to those which are perceived to be exactly the same. A direct comparison of the two adaptation responses revealed precisely this result (*t* = 2.05, *p* = .03), demonstrating that adaptation responses in early VC tracked subjective perception even when there was no physical change in the stimuli (Fig. 6C).

Although no MTL region displayed evidence of an overall scene adaptation effect, we nevertheless investigated whether the PHC and RSC might display a *differential* adaptation effect. Both regions displayed differential adaptation in line with the subjective perception of the scenes, showing less adaptation for scenes perceived to be different (collapsed across hemisphere: PHC *t* = 1.81, *p* = .04; RSC *t* = 1.7, *p* = .05). Thus, although these regions did not show a global adaptation effect in response to repeated scenes, they nevertheless showed the expected pattern of differential adaptation. These results, therefore, are broadly consistent with the results of Park et al. (2007), and suggest that both the PHC and RSC display activity that tracks the subjective perception of scenes. By contrast, the HC did not display a significant effect of adaptation (*t* = 1.43, *p* = .08).

### 3.6 HC–VC connectivity

**Fig. 7:**
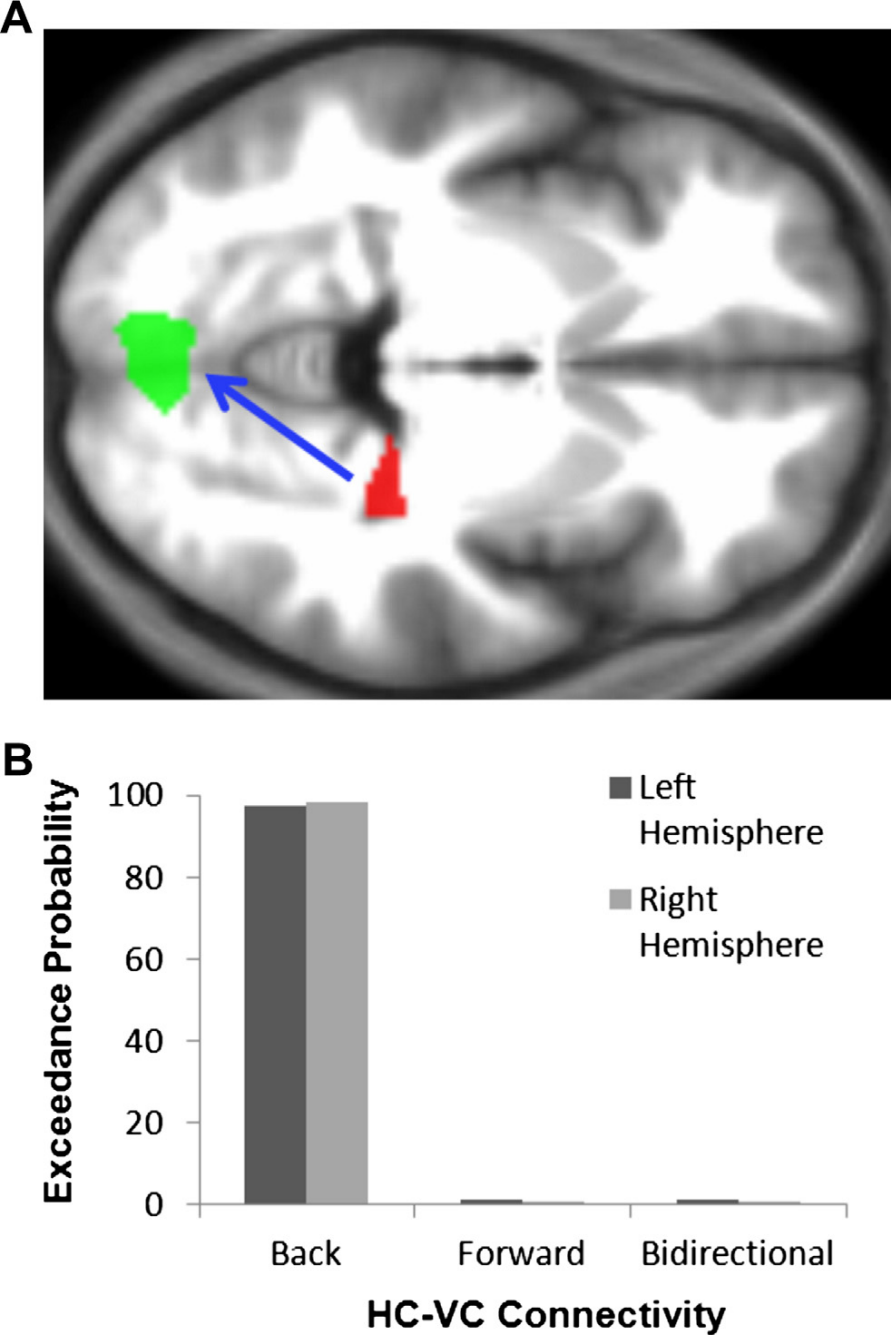
Modelling HC–VC connectivity. (A) The hypothesised flow of information, with activity in early VC being actively modulated by the HC during the BE effect. The HC is displayed in red and VC in green on an axial slice from the group average structural MRI scan. (B) The results of the DCM model comparison analysis. This plot displays the exceedance probability on the *y* axis, which describes how likely each model is compared to any other model. As hypothesised, the ‘back’ model was the clear winner. This suggests that the HC actively influences the updating of scene representations in early VC following BE.

Our results suggest that the MTL, and particularly the HC, was involved in the rapid, automatic extrapolation of scenes beyond the edges of the given view. For VC to show a differential adaptation response means that the subjective scene representations, including the extended aspects of scenes, must be made available to this region before the onset of the second scene *via* some top–down influence. In order to investigate this, and given the hippocampal results noted above, we applied a DCM analysis to the neural dynamics of the HC and early VC during the presentation of the first scene. If the HC was actively involved in updating the visual representations including the extended scenes in line with subjective perception, then we would expect to find evidence for modulation of VC activity by the HC on those trials where BE occurred. This model was compared to two alternative models (modulation of HC activity by VC, and bidirectional modulation). Backward modulation of VC by the HC was the winning model (exceedance probability of 97%), with robust results across both hemispheres (Fig. 7). These findings therefore confirm that activity in early VC was modulated by the HC when the BE effect occurred, and that this happened during or shortly after the initial stage of scene extrapolation.

## 4 Discussion

BE is an intriguing scene-specific phenomenon whereby people reliably remember seeing more of a scene than was present in the physical input, because they extrapolate beyond the borders of the original stimulus (Intraub and Richardson, 1989). By embedding the scene that is currently being viewed into a wider context, this supports the experience of a continuous and coherent world, and is therefore highly adaptive. Here we found that this extrapolation of scenes occurred rapidly around the time a scene was first viewed, and was associated with engagement of the HC and PHC. Notably, we found that the HC in particular seemed to drive the BE effect, exerting top–down influence on PHC and indeed as far back down the processing stream as VC. Subsequently, these cortical regions displayed activity profiles that tracked trial-by-trial subjective perception of the scenes, rather than physical reality, thereby reflecting the BE error.

### 4.1 Functional neuroanatomy of BE

BE is well-characterised cognitively (Intraub, 2012; Hubbard et al., 2010), but surprisingly little is known about its neural substrates. The only two previous neuroscientific studies of BE implicated different brain areas, the PHC and RSC in Park et al. (2007), and the HC in Mullally et al. (2012). Our results reconcile and extend these studies. By focussing specifically, and for the first time, on the initial stage of BE (the BE effect) the point of the extrapolation of scenes, we found that the HC was central to this process, in line with the results of Mullally et al. (2012) where focal bilateral hippocampal damage resulted in attenuated BE. The hippocampal response we observed was manifested rapidly during or just after the initial exposure to a scene and, importantly, before the second presentation of the scene. This confirms that hippocampal involvement was in the initial phase of extrapolating what was beyond the view rather than any subsequent memory-related effect. A DCM analysis showed that the HC influenced activity in PHC. When considered alongside the results of the adaptation analyses, where PHC, RSC and VC responded to the subjective perception of scenes, this indicates that these brain areas play a more active role in the second, BE error, phase of BE. This accords with the PHC and RSC findings of Park et al. (2007), where they specifically focussed on the BE error, and not the initial BE effect. Overall, therefore, our results serve to underscore the two-stage nature of BE whilst also characterising the underlying neuroanatomy associated with each phase.

### 4.2 The role of the HC

We next consider in more detail the role of the HC in the BE effect, and how this might provide insights into the nature of hippocampal processing. The HC is known to be involved in spatial navigation, recalling past experiences, and imagining fictitious and future scenes and events (Buckner and Carroll, 2007; Hassabis and Maguire, 2007; Addis and Schacter, 2011; Spreng et al., 2009). Hassabis et al. (2007) found that patients with selective hippocampal damage and amnesia were unable to construct and visualise fictitious and future scenes and events in their imagination (see also Klein et al., 2002; Hassabis et al., 2007; Rosenbaum et al., 2009; Andelman et al., 2010; Race et al., 2011). This led to the proposal that the HC supports scene construction, defined as the process of mentally generating and maintaining a complex and spatially coherent scene or event (Hassabis and Maguire, 2007, 2009). It was further argued that key functions such as episodic memory and spatial navigation may critically depend on scene construction (Hassabis and Maguire, 2007). In line with previous reports, the patients in Mullally et al.'s (2012) study with selective bilateral hippocampal damage and amnesia were also unable to explicitly construct and visualise scenes in the imagination. BE, which depends on the ability to construct coherent representations of scenes beyond the view, was also attenuated in these patients. This demonstrated the automatic and implicit role of the HC in scene construction. Our fMRI data corroborate and extend the results of Mullally et al. (2012) by now pinpointing that the precise contribution of the HC to BE is the initial, rapid extrapolation of scenes. That the intact PHC and RSC of Mullally et al.'s (2012) patients were unable to compensate for their damaged hippocampi and could not rescue BE, resonates with our finding of the HC being the driving force behind scene construction and BE, and subsequently influencing other areas such as PHC.

A key question that naturally arises is what exactly the HC does in the service of scene construction and BE? Constructing a scene in the mind's eye or imagining what might be beyond the view as in BE, involves a number of operations including being able to predict what is likely to be in a scene or beyond the view, accessing prior episodic and semantic knowledge relevant to that context, associating items together and with the scene context, and placing all this information in a coherent spatial framework. A possible clue about the specific role of the HC comes from the recent study of Mullally et al. (2012). Patients with hippocampal damage and amnesia were shown a scene and were able to describe it in great detail. When asked to imagine taking a step back from the current position and describe what might then come into view, the patients' performance was comparable to the control participants. They were able to anticipate with accuracy what would be beyond the view, list contextually relevant items in the extended scene, and could associate them with one another and with the context. However, in stark contrast to controls, the patients omitted spatial references almost entirely from their descriptions of what was likely to be beyond the view, a difference that was not apparent for the other scene elements. Moreover, they rated the extended scene as lacking spatial coherence. This is also true of attempts to imagine fictitious or future scenes in general, where amnesic patients' constructions were spatially fragmented (Hassabis et al., 2007; Mullally et al., 2012). Thus, one proposal is that the HC implements the spatial framework of scenes when they are not physically in view (Hassabis and Maguire, 2007, 2009). The posterior location of the hippocampal activations observed here in relation to the BE effect fit with a possible spatial role, as this region has been implicated in spatial navigation and memory in a range of contexts (e.g., Moser and Moser, 1998; Maguire et al., 2000; see also Poppenk and Moscovitch, 2011). Clearly more work is required to explore the link between scenes, space and the HC further, along with other accounts of its role in scene processing (Graham et al., 2010; Bird et al., 2012). Overall, however, what the scene construction and BE work highlights, and this is particularly evident in our current fMRI findings, is that the internal, automatic construction of scenes may be a central operation of the HC.

### 4.3 Adaptation and inter-regional connectivity

Using fMRI we were able to establish the brain areas supporting the highly adaptive BE effect, and in so doing to provide further evidence for the role of the HC in constructing unseen scenes. Another key advantage of fMRI that we exploited here is the ability to appreciate the distributed set of brain areas engaged by a task and, crucially, how these areas interact. As noted above, we found that two high-level scene-related areas, the PHC and RSC, both showed activity profiles that mapped onto subjective perception. This result suggests that these regions do not simply contain veridical representations of the physically presented scenes, but are actively updated to include information about extrapolated scenes beyond the boundaries of the physical scenes. Intriguingly, we found that early VC also displayed differential fMRI adaptation effects that reflected the subjective perception of the scenes. Specifically, VC showed greater adaptation when no change was perceived between two scene presentations, compared to those trials where the second scene appeared to be closer (consistent with the BE error). Importantly, the two scenes on each trial were always identical, so this effect cannot be attributed to any physical changes in the stimuli, and can only be due to a change in subjective perception driven by a top down process. This latter result is consistent with a variety of studies which have shown that activity as early as V1 can reflect changes in subjective perception (Tong, 2003; Kamitani and Tong, 2005; Murray et al., 2006; Sperandio et al., 2012), and we now demonstrate that this can also be the case with the processing of complex scenes.

It should be noted that Park et al. (2007) also looked for similar adaptation results within retinotopic cortex and failed to find any evidence for such an effect. The disparate findings are likely due to differences in the study designs. Specifically, Park et al. (2007) used an implicit task where inferences were made on the basis of different conditions which, on average, produced different degrees of the BE effect. By contrast, we recorded explicit trial-by-trial behavioural choice data, which allowed us to directly compare trials which individuals perceived as the same to those where BE occurred. This latter approach is likely to have provided substantially greater power to detect activity relating to subjective perception of scenes within early VC.

The relationship between the HC and this cortical network of regions was elucidated further by the DCM connectivity analyses. Put simply, DCM indicates the direction of flow of information, and which brain areas are exerting an influence on others. We found that activity within PHC and early VC was influenced by the HC. This modulation suggests that the scene representation within PHC and VC is actively updated by a top–down connection from the HC to represent the extended scene. This updated (subjective) representation then leads to the subsequent differential adaptation effect. That the studied scene need only be absent for as little as 42 msec for BE to be apparent (Intraub and Dickinson, 2008), underscores the rapidity of this modulatory process.

Put together, our BE findings offer a new insight into the neural basis of scene processing. They suggest a model whereby the HC is actively involved in the automatic construction of unseen scenes which are then channelled backwards through the processing hierarchy *via* PHC and as far as early VC in order to provide predictions about the likely appearance of the world beyond the current view. This subsequently leads to a differential adaptation effect within early VC which is driven by a subjective difference in appearance due to the extended boundaries. The fact that the information about the extended scene is automatically and rapidly conveyed as far back as early VC suggests that anticipation and construction of scenes is a pervasive and important aspect of our online perception, with the HC playing a central role.
